# Effects of Exercise on Memory Consolidation and Retrieval of Passive Avoidance Learning In Young Male Rats

**DOI:** 10.5812/asjsm.34858

**Published:** 2010-09

**Authors:** Hakimeh Saadati, Shirin Babri, Naser Ahmadiasl, Mohammad Mashhadi

**Affiliations:** 1 Department of Physiology, Ardabil University of Medical Science, Ardebil, IR Iran; 2 Neuroscience Research Center, Tabriz University of Medical Sciences, Tabriz, IR Iran; 3 Department of Physiology, Faculty of medicine, Tabriz University of Medical Sciences, Tabriz, IR Iran; 4 Healthy Center, Ardabil University of Medical Sciences, Ardebil, IR Iran

**Keywords:** Exercise, Memory, Avoidance learning, Rats, Physical activity

## Abstract

**Purpose:**

Previous studies have shown that physical activity improves learning and memory. Present study was performed to determine the effects of short term and long term treadmill exercise on learning, memory consolidation and retrieval of passive avoidance learning in an animal model.

**Methods:**

In this study fifty male Wistar rats with 3-4 months of age were randomly divided into five groups (n=10 in each group). Control group was trained in passive avoidance box and was tested 10 min, 24 hr, 10 days and 3 months later. Two groups exercised on treadmill one hour at 17 m. min for 10 days and 3 months respectively and then were trained in passive avoidance box and were tested 10 min and 24 hr later. The other two groups were trained and were tested 10 days and 24 hr later and then exercised on treadmill as same as other exercised groups.

**Results:**

Obtained results showed that short-term (10 days) and long-term (3 months) treadmill running before training by passive avoidance test had significant (P=0.006 and P=0.001 respectively) effects on memory consolidation. However, no significant difference was observed between latency time of rats before and after exercise in exercised groups retrieval (P>0.05).

**Conclusion:**

Our results showed that physical activity promoted learning and memory consolidation but it did not affect retrieval memory performance.

## INTRODUCTION

Studies have revealed that maintaining brain health is an important public health goal, which physical activity or exercise can help us to achieve^[1]^. Recently, many investigations have been conducted to examine the influence of exercise on cognitive functions and consequently, several biological mechanisms have been suggested to explain the effects of exercise on learning and memory. Human and animal studies suggest that exercise retards aging, prevents from age-related diseases and increases the life expectancy^[2]^. Exercise has also beneficial effects on brain functions including plasticity promotion and learning and memory enhancement. These data have indicated that exercise leads to changes at the level of a number of gene transcripts known to be associated with the neuronal activity, synaptic structure and synthesis of neurotransmitters that are important in memory processing^[3]^.

Studies showed that voluntary exercise induces brain derived neurotrophic factor (BDNF) mediated mechanism that promotes neuroplasticity^[4, 5]^. Other trophic factors, including nerve growth factor (NGF), fibroblast growth factor 2 (FGF2), insulin-like growth factor I (IGF-1) and vascular endothelial growth factor (VEGF) levels were also induced in response to exercise and mediated growth and differentiation of neurons^[6,7,8]^.

Findings have suggested that adult brain continues to generate new neurons in response to exercise in the hippocampus^[9, 10]^. Hippocampus receives information from each of the sensory modalities and projects widely throughout the brain^[11]^.This area is best known for its role in learning and memory^[12]^. Exercise has also been shown to enhance hippocampus cholinergic functioning^[13]^. Several studies have suggested that exercise influences the levels of the amines and endorphins in the body and these changes can have a positive influence on cognitive functions. Thus, higher levels of norephinephrine, catecholamine, serotonin and other neurotransmitters might have contributed to the beneficial effects of exercise on cognitive performance abilities^[1, 6]^.

Samorajski and et al have found that exercise increased latency in middle and old aged significantly and has the lowest effect on adult mice^[2]^. Study of Radak et al. have showed that middle aged exercised rats had a significantly better short-term and long-term memory than age matched control rats^[14]^. Conversely, Radak et al conducted another case-control study, in which exercise did not significantly alter the memory retrieval and latency compared with control group^[15]^. Therefore, the main objective of this study, was to determine the effects of treadmill running on memory consolidation and retrieval.

## METHODS AND SUBJECTS

**Experimental animals:** Fifty male 3-4 months of age Wister rats, weighing 250±50gr, were obtained from our laboratory breeding stock housed singly under controlled conditions [temperature 22±2 c, relative humidity 40–70% and light/darkness cycle 12/12 h (lights on at 8:00 a.m.)]. Food and water were available in addition to libitum. The measurements were always done during the first half of the light cycle. Rats were pre-tested to determine their treadmill running willingness and those rats, which refused to run, were excluded before the start of experiments.

Rats were divided into five groups as follows:

1. One control group, which was trained in passive avoidance box, and was tested 10 min, 24 hr, 10 days and 3 months later.
2. Exercised – two groups (with exercised before training) that exercised on treadmill for 10 days and 3 months respectively and then were trained in passive avoidance box and were tested 10 min and 24 hr later.
3. The other two groups were trained first and were tested 10 days and 24 hr later and then exercised on treadmill.

**Exercise:** All exercised groups of animals were familiarized to treadmill apparatus in order to eliminate stress of exercise. These groups ran on treadmill for one hour at the speed 17m.min (-1) every day. The exercise workload consisted of running at the speed of 5m.min for the first 5min, 10m.min for the next 5min and then 17m.min for last 50 min with 0 degree ^[16, 17]^. All animals tolerated the speed and duration of the exercise and completed successfully the training.

**Retention of passive avoidance learning response:** The retention of passive avoidance learning response behavior was investigated in a multi trial step-through paradigm. The apparatus consisted of two compartments.One being dark and the other a well-lit white area (30 /21/20 cm3).A small sliding door separated the two compartments.

All experimental groups had the first 10min as adaptation. They were allowed in the dark compartment to be habituated to the apparatus. The rat was placed in the illuminated compartments and 5 s later the guillotine door was raised. The habituation trial was repeated after 30 min and at the same interval. After third trial a strong electric foot shock (1mA, 5s) was delivered to the dark box via the stainless steel bars, which served as the floor. Training was terminated when the rat remained in the light compartment for 120 consecutive seconds. But the rat was retained in the apparatus and received a foot- shock each time if reentered the dark compartment (multi trial). Latency of entering into the dark compartment was recorded after 10min, 24 h, 10 days and 3 months in control and exercised groups ^[14, 18]^.

**Statistical analysis:** The results of the passive avoidance tests in two exercised groups were compared using ANOVA test with Tukey's post-hos for assessing memory consolidation. Besides, the results of the passive avoidance tests in two exercised groups were compared with each other by paired t test for assessment of memory retrieval. A P value of <0.05 was taken statistically significant. All data were expressed as mean ± standard error of the mean (S.E.M).

## RESULTS

Ten days and three months exercise did not result in significant differences between exercised and control groups. Treadmill running of the rats induced a significant enhancement to the passive avoidance learning and memory consolidation.

The time passed for a rat to enter the dark box (latency time) was determined: 471 ± 65.5sec in short-term (10 days) and 521 ± 42sec in long-term (3 months) exercised groups. The analysis indicates that short term and long term have significant (P=0.006 and P=0.001 respectively) effects on learning and memory consolidation in comparison with control group (155±75sec).

**Fig. 1:**
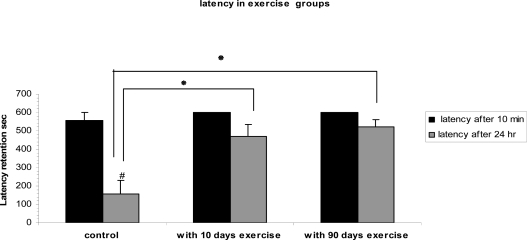
Effects of treadmill exercise on memory consolidation: Short-term and long-term exercised groups showed significant increases latency time compared to the control group (P=0.006 and P=0.001 respectively). Values are mean ± S.E.M for 10 animals per group. # P<0/001 compared to latency time after 10 min in control group

Comparison of the delay times between short term and long term exercised groups showed no significant difference between these groups (Fig. 1). In other exercised groups for assessing the effect of exercise on the memory retrieval, latency time in 10 days (after short-term exercise) and 3 months (after long-term exercise) after training in comparison with latency time before exercise (24 hr after training) were not significantly (P>0.05) different. Therefore in the present study treadmill exercise did not promote memory retrieval (Table 1).

**Table 1:** Effects of long term (3 months) and short-term physical activity on memory retrieval in passive avoidance test

Latency time in experimental groups with exercise after training			
Short term (10 days) exercise		Long term (3 months) exercise	
After exercise	Before exercise	After exercise	before exercise
119±31	201±32	106±31.3	186±80.2

Values are mean±S.E.M for 10 animals per group. A value of P<0.05 was considered to be significant

Latency time at different time intervals [24 h (155±75 sec), 10 days (107±56 sec) and 3 months (86.1±25.2 sec)] did not show any statistically significant difference in control group.

## DISCUSSION

Although the results of animal experiments need to be confirmed in human studies, results of the present study, which emphasizes the importance of physical activity as a life style, have a potentially great value in public health. There are a variety of potential mechanisms that can cause the exercise induced learning and memory improvements. Positive effects of environmental enrichment on memory have previously been shown. Over the past decade, a number of studies have shown the benefits of exercise on brain health and its function, particularly in aging populations^[2, 19, 20]^.

Our findings showed that short-term (10 days) and long-term (3 months) treadmill exercise before training enhanced latency time of adult rats in passive avoidance learning and memory consolidation performance significantly. Nevertheless, no significant difference was observed between long term and short term exercise in the passive avoidance test.

Different factors may be mediated the beneficial effects of exercise on cognitive functions and brain. Studies suggest that physical exercise is a behavioral intervention to enhance neurogenesis and plasticity in the hippocampus^[6, 9, 10, 21, 22]^. In addition, exercise can increase neurotrophic factors^[4, 5]^, neurotransmitters and growth factors. Another effect of exercise is enhancement of non neural components of brain, such as vasculature^[6,7,8]^. Other studies have shown that long-term potentiation (LTP) and memory function are elevated after exercise^[2, 9, 17]^ and facilitate recovery from traumatic brain injury in mediating the exercise – induced enhancement in brain derived neurotrophic factors (BDNF)^[10, 23,24,25]^. These factors support the hypothesis that treadmill exercise can increase learning and memory consolidation in young rats.

We did not find any effect of treadmill running on memory retrieval. Indeed, passive avoidance test showed that after exercise latency time was not significantly different from latency time of before exercise. Another study demonstrated that exercise at a higher – intensity level (20-25 m/min for 20 min) did not affect learning. Increased levels of exercise (25 m/min for 25min) corresponded with an early impairment of spatial memory^[26]^. Another research has revealed that exercise can facilitate acquisition, but has not affect performance following acquisition^[27]^. Results of another study have reported that 14 days of exercise increased the rate of acquisition in the Y maze also is beneficial for the retrieval of spatial reference memory^[21]^.

Another investigation has indicated that exposure to emotionally arousing events may facilitate the consolidation or storage of new information, and simultaneously impair the recall of previously learned information^[28]^. Different factors may be mediated the beneficial effects of exercise on cognitive functions and brain. Further research needs to be conducted to identify neurotransmitters and other factors in male and female animal models as well as human.

## CONCLUSION

Our results suggest that treadmill running improved learning and memory consolidation of rats but it had not any significant effect on memory retrieval.
